# Identification of Fur in *Pasteurella multocida* and the Potential of Its Mutant as an Attenuated Live Vaccine

**DOI:** 10.3389/fvets.2019.00005

**Published:** 2019-02-04

**Authors:** Qing Liu, Yunlong Hu, Pei Li, Qingke Kong

**Affiliations:** ^1^College of Animal Science and Technology, Southwest University, Chongqing, China; ^2^Institute of Preventive Veterinary Medicine, College of Veterinary Medicine, Sichuan Agricultural University, Chengdu, China

**Keywords:** *Pasteurella multocida*, fur, iron uptake, attenuated live vaccine, immune responses

## Abstract

*Pasteurella multocida* is a pathogenic microorganism that causes a variety of serious diseases in humans and animals worldwide. The global regulator gene, *fur*, plays an important role in pathogenesis and regulates the virulence of many bacteria. Here, we identified a *fur* gene in *P. multocida* by complementing a *Salmonella* Choleraesuis Δ*fur* mutant, and characterized a *fur* mutant strain of *P. multocida*. The *P. multocida* Δ*fur* mutant strain exhibited no significant differences in growth and outer membrane protein (OMP) profiles when the complemented strain was compared to the parent. Ducks were used as the model organism to determine the virulence and protection efficacy induced by Δ*fur* mutant strain. Animal experiments showed that colonization by the mutant was decreased by oral infection of live Δ*fur* mutant strain. The LD_50_ of the ducks infected with the Δ*fur* mutant was 146-fold higher than that of the ducks infected with the wild-type strain when administered through the oral route. Evaluation of the immunogenicity and protective efficacy of the Δ*fur* mutant of *P. multocida* revealed strong serum IgY and bile IgA immune responses following oral inoculation with the Δ*fur* strain. Ducks that were orally inoculated with the Δ*fur* mutant strain demonstrated 62% protection efficacy against severe lethal challenge with the wild-type *P. multocida*. This study provides new insights into *P. multocida* virulence and the potential use of an attenuated vaccine against *P. multocida*.

## Introduction

Iron is an essential limiting element in bacterial metabolism, and its availability plays a vital role in the pathogenesis of most bacteria (1). Many enzymes require iron as a necessary co-factor for a wide variety of physiological processes (2), such as respiration, DNA biosynthesis, gene regulation, and the tricarboxylic acid (TCA) cycle (3). Proteins such as iron-containing heme and iron-sulfur cluster proteins act not only as excellent electron carriers but also as environmental or intracellular sensors that regulate gene expression (4). However, excess iron is highly toxic to cells. In particular, iron is an enhancer of oxygen toxicity because this metal efficiently catalyzes the Fenton reaction, which converts hydrogen peroxide to the highly reactive hydroxyl radical (5). Therefore, during the process of iron uptake, bacteria maintain a dynamic balance of iron ions within an ideal range by adjusting rates of uptake. Precise regulation of the intracellular iron concentration is typically closely associated with iron acquisition genes, which are controlled via the ferric uptake regulator (Fur) protein. The Fur protein is a metal ion-dependent transcription factor that is ubiquitously expressed in bacteria and controls the transcription and expression of genes involved in a variety of cellular functions, including iron uptake (6). Fur binding to metal ions causes a conformational change in the regulator, which promotes and stabilizes its interaction with DNA (5). Fur-Fe^2+^ complexes bind to the DNA promoter regions (Fur boxes) of genes involved in iron acquisition, resulting in transcriptional repression or activation (7). In certain bacteria, Fur regulates biofilm formation (8–10) and plays an important role in disease pathogenesis, host-bacteria interactions that control virulence factor expression, and the *in vitro* regulation of toxin production. The absence or impairment of the *fur* gene induces decreased virulence of *Staphylococcus aureus, Listeria monocytogenes*, and *Vibrio cholerae* in mouse models (11–14). Moreover, in fish models, an *Aeromonas salmonicida fur* mutant exhibited significantly reduced virulence compared to the wild-type strain, demonstrating its potential for use as a live-attenuated vaccine (1). *Pasteurella multocida* is a Gram-negative, capsulated, non-motile, highly common bacterium that infects a range of animals through respiratory or oral routes (15), resulting in significant economic losses to industries worldwide (16). Currently, these bacteria are divided into 5 serotypes (A, B, D, E, and F) based on capsular typing and further classified into 16 serotypes (1–16) according to the type of lipopolysaccharide (LPS); different serotypes exhibit varying degrees of virulence in animals (17). Due to the lack of effective coverage in multi-serotype vaccines, antibiotics remain the most commonly employed treatment for avian cholera despite their correlation to increased drug resistance and food safety risks of contaminating bacteria (18). Furthermore, existing live-attenuated vaccines have been observed to revert to virulent strains (19). Therefore, the development of an efficient and safe live-attenuated vaccine for cholera control in fowl is urgently needed. In the present study, we characterized the *P. multocida* Fur protein. Specifically, the previously putative *fur* gene of *P. multocida* was identified through heterologous complementation of *P. multocida fur* in a *Salmonella* Choleraesuis Δ*fur* mutant. A Δ*fur* mutant of *P. multocida, P. multocida* 0818, isolated from a duck was produced. We investigated several phenotypes of the wild-type and Δ*fur* mutant strains including growth curves, outer membrane protein (OMP) profiles, tolerance to polymyxin B and bile salt deoxycholate (DOC), and serum sensitivity. We also examined virulence colonization levels and immunogenicity of *fur* mutant in a natural host, the Sheldrake duck.

## Materials and Methods

### Bacterial Strains, Plasmids, Media, Growth Conditions, and Reagents.

The bacterial strains and plasmids used in the present study are listed in Table 1. The *P. multocida* 0818 strain was isolated from a pasteurellosis outbreak at a duck farm in Sichuan Province, China in 2012 (21). Because the genome of *P. multocida* 0818 has not been sequenced, all of the primers designed for *P. multocida* 0818 in Table 2 were based on the genomic sequence of *P. multocida* Pm70 (AE004439.1) (25). *P. multocida* was cultured in Bacto brain heart infusion (BHI) broth (BD Bioscience, San Jose, CA, USA), and *Salmonella* Choleraesuis and *Escherichia coli* were grown in Luria-Bertani (LB) broth. The antibiotics and reagents used in this study were purchased from Sigma-Aldrich (St. Louis, MO, USA). LB broth, BHI broth, and chromoazurol S (CAS) broth were routinely used (26). When required, the media were supplemented with 1.5% agar, kanamycin (Kan) (50 μg/ml), or chloramphenicol (Cm) (25 μg/ml for *S*. Choleraesuis and *E. coli*, 2.5 μg/ml for *P. multocida*) and ampicillin (Amp) (100 μg/ml). Diaminopimelic acid (DAP) was added to the LB broth (50 μg/ml) for *E. coli* growth because the χ7213 strain needs DAP to compensate for the Δ*asd* mutation. Bacterial growth was spectrophotometrically detected at a wavelength of 600 nm.

**Table 1:** Bacterial strains and plasmids used in the present study.

**Strain or plasmid**	**Description**	**Source**
**STRAINS**		
S340	*S*. Choleraesuis	(20)
S984	*S*. Choleraesuis Δ*fur*	This work
*P. multocida* 0818	Wild-type *P. multocida* serotype A strain. Capsulated and virulent	(21)
S985	*P. multocida* 0818 Δ*fur*::*kanR*	This work
χ7232	*E. coli* K-12, *endA1 hsdR17* (r_K_-, m_K_+) *glnV44 thi-1 recA1 gyrA relA1* Δ(*lacZYA-argF*)*U169 λpir deoR* (φ*80dlac* Δ(*lacZ*)*M15*)	(22)
χ7213	*E. coli* K-12, *thi-1 thr-1 leuB6 glnV44 fhuA21 lacY1 recA1 RP4-2-Tc*::Mu λ*pir* Δ*asdA4* Δ*zhf-2*::Tn*10*	(22)
**PLASMIDS**		
pET-32a-*fur*	For the expression of *P. multocida* Fur	This work
pSS664	Asd^+^ P_trc_ pSC101 origin replication p15a, *asd*^+^, spec^r^	Derived from pYA3337
pYA4807	Deletion of *fur* gene of *Salmonella*	(6)
pSS908	Insertion of *fur* into pSS664	This work
pYA4278	pRE112 derivative, *sacB mobRP4* R6K *ori* Cm^+^	(23)
pSS909	pYA4278-Δ*fur*	This work
pSS910	pYA4278-Δ*fur*::*kanR*, for deletion of *fur* in *P. multocida*	This work
pMC-Express	Broad-host-range shuttle vector derived from pMIDG100, chloramphenicol^r^	(24)
pSS911	Insertion of *fur* into pMC-Express	This work

**Table 2:** Primers used in the present study.

**Primers**	**Sequence 5^**′**^ - 3^**′**^**
C*fur*-F	CGCGGATCCTGTCTGAAGAAAATATCAAAC
C*fur*-R	CCCAAGCTTTTATTTTTTGCCATTCTCATCG
pET-*fur*-F	GGGAATTCCATATGTCTGAAGAAAATATCAAAC
pET-*fur*-R	CGCGGATCCTTAGTGGTGGTGGTGGTGGTGTTTTTTGCCATTCTCATCG
P*fur*-F	cgcGGATCCatgTCTGAAGAAAATATCAAAC
P*fur*-R	ATAAGAATGCGGCCGCTTATTTTTTGCCATTCTCATCG
D*fur*-1F	CTCATGATTGGGATTCCAAC
D*fur*-1R	CCTGCAGGGATGCGGCCGCAATAGCCCCTTAATTATTTTGC
D*fur*-2F	GCGGCCGCATCCCTGCAGGAAACAAGGCGAATTAAATAAAAAG
D*fur*-2R	ATTTTCCTTGATTGACTGGCTTGACACCATGAGTGATGAAG
*Kan*-F	ATAAGAATGCGGCCGCTCAGTGGAACGAAAACTC
*Kan*-R	CCTGCAGGTTAGAAAAACTCATCGAGCATC
Primer 1	ATGTCTGAAGAAAATATCAAAC
Primer 2	TTATTTTTTGCCATTCTCATCG
Primer 3	CCATCATTGTTGGTCACTGG
Primer 4	CAATATTTTCACCTGAATCAG
Primer 5	CTGATTCAGGTGAAAATATTG
Primer 6	GTCACGAAGTTGAGCAAAC
16sRNA-F	TAATACCGCGTATTCTCTGAGG
16sRNA-R	CCCTCCCTAAAGTACTCTAGAC

### Molecular and Genetic Procedures

Restriction enzymes were obtained from New England BioLabs (Ipswich, MA, USA). PrimeSTAR Max DNA polymerase (Takara Bio Inc., Shiga, Japan) and Taq DNA polymerase (Vazyme Biotech Co., Ltd., Nanjing City, China) were used in all PCR assays. The primers employed in the present study are listed in Table 2. When required, a PCR purification kit and a Plasmid Mini Preparation Kit purchased from Tiangen Biotech (Beijing, China) were used, and the commercial sequencing was performed at BGI Tech (BGI Tech Solutions Co., Ltd., Shenzhen, China).

### Detection of Secreted Siderophores

The *fur* gene of *P. multocida* 0818 was amplified and cloned into the low-copy-number plasmid pSS664 using two primers, C*fur*-F/C*fur*-R, which contained BamHI and HindIII cleavage sites, resulting in the recombinant plasmid pSS908. This plasmid was transformed into a *Salmonella* Choleraesuis Δ*fur* mutant to generate the heterologous expression strain S984 (pSS908). Because the Fur protein is a global regulator, which negatively regulates iron uptake-related genes involved in siderophore production in *Salmonella*, the siderophore activity will reflect the status of Fur synthesis in *Salmonella*. Therefore, detecting siderophore production will be indicative of Fur synthesis in *Salmonella* (27). The production of siderophores was detected using chemical assays as previously described (26). The approach was based on the ability of a siderophore with a high affinity for iron (III). An indicator dye with chrome azurol S (CAS) and hexadecyltrimethylammonium bromide serves as an indicator. The CAS indicator solution consists of 60.5 mg of Chrome Azurol S, 27 mg of Ferric Chloride (FeCl_3_·6H_2_O), and 83.3 μl of concentrated hydrochloric acid (HCl) in 200 ml H_2_O. The color of this indicator dye is blue in the presence of iron (III); however, when a strong chelator, such as siderophore, removes the iron from this dye, it turns from blue to yellow/orange(26). Fifty milliliter CAS indicator solution was added to 1 L minimal basal media agar to make CAS indicator plates (26). The assays were performed by streaking the bacterial strain grown under iron-limiting culture on the CAS agar plate, after overnight inoculation (about 18 h) in 37°C condition, a yellow-orange around the bacterial colony is visualized, indicating bacterial siderophore production, which is negatively regulated by Fur, and the observed blue color around bacterial indicates absence of siderophore.

### Expression and Purification of Fur Protein and Generation of a Polyclonal Mouse Anti-Fur Antibody

To obtain Fur protein, the *fur* gene was amplified from *P. multocida* 0818 genomic DNA using two primers, pET-*fur*-F/pET-*fur*-R, which contained BamHI and NdeI enzyme cleavage sites and six histidine tags in the C-terminus. The complete *fur* fragment was amplified, then digested using the aforementioned restriction enzymes, and subsequently inserted into the BamHI-NdeI-digested linear pET-32a expression vector, generating pET-32a-*fur*. The genetic elements of the recombinant plasmid, pET-32a-*fur*, were confirmed through sequencing and subsequently transformed into competent *E. coli Rosetta* cells with ampicillin selection. Fur protein expression was induced using different concentrations of isopropyl β-D-thioglucopyranoside (IPTG; Sigma-Aldrich, St. Louis, MO). The Fur protein was characterized by 12.5% SDS polyacrylamide gel electrophoresis (SDS-PAGE). Subsequently, 6 × His/Ni-NTA affinity chromatography was employed for Fur protein purification following the product procedure. To prepare a polyclonal mouse anti-Fur antibody, five female Kunming mice were immunized twice at a 2-week interval through intramuscular injection with the Fur protein (1 mg) and QuickAntibody-Mouse3 W adjuvant (Bio Dragon Immunotechnologies Co., Ltd., Beijing, China). Subsequently, a blood sample was collected from the eye socket, and mouse antisera were prepared 3 weeks after the second immunization. This antibody was used for detecting Fur expression in the wild-type strain, the Δ*fur* mutant S985, and S985 (pSS911) using the western blotting procedure was performed as previously described (28).

### Construction of a *P. multocida fur* Deletion Strain

To construct a *fur* deletion mutant strain, 500-bp upstream and downstream fragments of the *fur* gene were amplified using *P. multocida* 0818 genomic DNA as a template, with the following primers: D*fur*-1F/D*fur*-1R and D*fur*-2F/D*fur*-2R (Table 2), respectively. The primers D*fur*-1R and D*fur*-2F contained NotI and SbfI enzyme cleavage sites. The fused DNA fragment was obtained by amplifying the upstream and downstream fragments using a PCR with the D*fur*-1F/D*fur*-2R primers. The suicide plasmid T-vector pRE112 was digested with AhdI to generate a linear T-vector (23), and the fused fragment was subsequently ligated into this T-vector, followed by the terminal addition of a polyA tail using the Tailing-A Reaction Kit (Tiangen Biotech) to generate the plasmid pSS909. Subsequently, the *KanR* cassette sequence, which is 905 bp in length, was amplified by PCR using the primers *Kan*-F/*Kan*-R and the plasmid pET28a as a template, followed by a restriction enzyme digestion with NotI and SbfI. The resulting DNA fragment was inserted into pSS909 to construct the recombinant plasmid pSS910. pSS910 was subsequently transformed into *E. coli* χ7213 and mobilized into *P. multocida* 0818 through intergeneric transfer using a filter mating method (21). Single crossover integrates were selected after growth on BHI agar containing 50 μg/ml Kan without DAP (to select against *E. coli*). Successful integration was confirmed by PCR analysis of genomic DNA from the *fur* mutant strain (21). The *fur* gene of *S*. Choleraesuis was deleted by suicide plasmid pYA4807 following the standard method in our lab (29), the resulting mutant strain was named as S984.

To construct complementary *fur* mutant strains in *P. multocida*, the *fur* gene sequence (441 bp in length) was amplified from *P. multocida* 0818 genomic DNA using two primers, P*fur*-F/P*fur*-R, which contain BamHI and NotI cleavage sites. The *fur* fragment was cloned into the pMC-Express plasmid (kindly gifted from Paul R. Langford at Imperial College London) through enzymatic digestion to construct the recombinant plasmid pSS911, and the resulting plasmid was introduced into the Δ*fur P. multocida* strain to generate complementary strains by electroporation.

### Phenotype Characterization

The cell growth curve for *P. multocida* 0818 was analyzed after recording the OD_600_ value every 2 h over a 24 h period. The OM proteins of *P. multocida* and *S. Choleraesuis* were prepared as previously described (20, 21). Briefly, BHI growth medium was used to grow 200 mL of cells that were harvested after reaching an OD_600_ of 0.8. Following repeated freeze-thaw cycles, the cells were then centrifuged and washed once in HEPES buffer (10 mM, pH 7.4) and re-suspended in 20 ml of 4-(2-Hydroxyethyl) piperazine-1-ethanesulfonic acid buffer (HEPES buffer) (10 mM, pH 7.4) on ice. Subsequently, the cells were disrupted through sonication (six bursts, 10 s each), and the intact cells were removed through centrifugation (15,600 × g, 5 min, 4°C) (21). The supernatants containing OMPs were transferred to new tubes and centrifuged again (15,600 × g, 30 min, 4°C). The membrane pellets were re-suspended in 2 ml of HEPES buffer. To solubilize the cytoplasmic membrane, 2 ml of 2% sarcosyl was added followed by incubation at room temperature (RT) for 30 min with constant shaking. After centrifugation (15,600 × g, 30 min, 4°C), the pellets containing the OMPs were washed once with 0.5 ml of HEPES buffer and re-suspended in 50 μl of HEPES buffer. The OMP concentrations were measured using the BCA Protein Assay Kit (Thermo Scientific, Rockford, IL, USA). Protein samples were then supplemented with a sample buffer [50 mM Tris, 20% glycerol, 4% SDS, 0.005% bromophenol blue, and 5% β-mercaptoethanol] and boiled at 95°C for 5 min. Subsequently, the samples were assessed by 12.5% SDS-PAGE and stained with Coomassie Brilliant Blue R-250 (Sigma-Aldrich).

### Polymyxin B and Deoxycholic Acid (DOC) Resistance Assays

Polymyxin B and DOC resistance assays were performed as previously described (30) with slight modifications. Cells were harvested from BHI broth and washed in PBS when *P. multocida* growth reached an OD_600_ of 0.8. The cells were subsequently suspended in BHI medium. The bacterial suspension was diluted to 1 × 10^6^ colony-forming units (CFU) per 100 μl and mixed with polymyxin B (0.5 μg/ml), DOC (250 μg/ml) solution, or untreated, followed by an incubation at 37°C for 1 h. Subsequently, the bacteria were 10-fold serially diluted with BHI medium, and diluted suspensions were spread onto BHI agar plates to determine the number of CFU per 100 μl. The survival rate was calculated as mean CFU of polymyxin B-treated or DOC-treated culture/mean CFU of an untreated culture. All tests were performed in triplicate.

### Determination of LD_50_ in Ducks

All animal experiments in this study were performed in strict accordance with the recommendations delineated in the Guide for the Care and Use of Laboratory Animals of the Ministry of Science and Technology of China. All animal procedures were approved by the Animal Ethics Committee of Sichuan Agricultural University (approval no. 2015-015). Ducklings used in the animal experiments were negative for *P. multocida* based on a serological assay. One-day-old Sheldrake ducks were purchased from a hatchery and acclimated for 7 days prior to the experiment. The *P. multocida* 0818 wild-type and Δ*fur* strains were grown in BHI media, then harvested and re-suspended in PBS when the cultures reached an OD_600_ of 0.8–0.9. The suspensions were subsequently diluted into various concentration doses (1 × 10^5^ CFU to 1 × 10^8^ CFU per 100 μl of PBS). Groups of 1-week-old ducks (10–15 ducks per group) were orally infected with 100 μl of PBS containing different doses of *P. multocida* 0818 or S985 (Δ*fur*), followed by observation for 2 weeks; the deaths were recorded daily.

### Colonization of Sheldrake Tissues by *P. multocida*

Colonization of the liver, spleen, and lungs by *P. multocida* in ducks was evaluated as previously described (23). Two groups of 1-week-old Sheldrake ducks (5 ducks per group) were orally infected with 100 μl of PBS containing ~1 × 10^8^ CFU of the *P. multocida* parent strain or the Δ*fur* strain. All animals were euthanized 3 days later, and 0.1-g samples from each of the selected organs of the infected ducks were removed by dissection. The dissected organ samples were placed in sterile micro-sample bags containing 900 μl of BSG and ground into uniform particles. Subsequently, the homogenates were 10-fold serially diluted, and 100 μl of the diluted suspensions were plated onto agar plates containing BHI or BHI supplemented with Kan.

### Serum Antibacterial Test

Duck sera were prepared from healthy adult ducks. The levels of serum antibacterial activity against the *P. multocida* strains were determined as previously described (31). *P. multocida* was grown in BHI media to an OD_600_ of 0.8, harvested and re-suspended in PBS, and the bacterial suspension was diluted to a concentration of ~1 × 10^4^ CFU/ml. An aliquot of 100 μl containing 1 × 10^4^ CFU/ml bacterial suspension was added to 900 μl of duck serum or heat-inactivated duck serum (heated at 56°C for 30 min). One hundred-microliter serum/bacterial mixtures were subsequently 10-fold serially diluted with PBS, and 100 μl of the diluted suspensions were spread onto BHI agar plates (supplemented with or without Kan). The remaining mixtures were cultured at 37°C for 3 h with shaking. Subsequently, the samples were diluted and spread using the same method. Viable counts were determined at 0 and 3 h. The growth rate was calculated as the average CFU/ml at 3 h divided by the average CFU/ml at 0 h. All tests were performed in triplicate.

### Immunization and Challenge of Sheldrake Ducks

Sichuan Sheldrake ducks were challenged using a previously described method (21). One-day-old Sheldrake ducks were purchased from a hatchery and acclimated for 7 days prior to the experiment. The ducks were fasted for 6 h prior to oral inoculation with the *P. multocida* strains and were not fed until 30 min after inoculation. Groups of 65 1-week-old ducks were orally immunized with 100 μl of PBS containing 1 × 10^5^ CFUs of the Δ*fur* strain on day 8 and boosted with the same dose of Δ*fur* on day 18. One set of controls was used: 30 1-week-old ducks that were orally inoculated with 100 μl of PBS. Those ducks that survived 28 days after oral inoculation were challenged with 1 × 10^8^ CFUs of the *P. multocida* 0818 wild-type strain (500 times 50% lethal doses [LD_50_]). The ducks were fed twice daily with duckling feed without antibiotics and followed by observation, and deaths were recorded daily. Moribund ducks were euthanized and subsequently necropsied to evaluate the presence of *P. multocida* in the liver, spleen, and lungs.

### Enzyme-Linked Immunosorbent Assay (ELISA)

Blood and bile samples were collected from each group on day 0 (prior to immunization), day 10 (before booster immunization), and day 20 (prior to challenge) as previously described (32). Five randomly selected ducks in the oral immunization group and control group were euthanized separately and then subjected to individual sampling. The *P. multocida* 0818 wild-type strain was harvested when the culture reached an OD_600_ of 0.8 in BHI media. A total of 1 × 10^10^ CFUs of *P. multocida* was obtained by centrifugation, washed, and suspended in PBS. Then, 1 × 10^10^ CFUs of whole-cell antigens of *P. multocida* 0818 were inactivated after heating at 80°C for 10 min. Subsequently, heat-inactivated whole-cell antigens of *P. multocida* or 0.25 μg/ml purified *P. multocida* OMP were independently applied to each well of a 96-well ELISA microtitre plate (Nunc-immuno MaxiSorp plate, Nunc, Roskilde, Denmark), both in a volume of 100 μl per well. The plates were then incubated at 4°C overnight, washed three times with 200 μl of PBST per well, and blocked with 2% BSA diluted in PBS (1 h at room temperature). The serum and bile samples from immunized and non-immunized ducks were then diluted to 1:160 and 1:40, respectively, and 100-μl aliquots of these dilutions were added to each well. After incubation for 1 h at 37°C, the samples were washed five times with PBST, and 100 μl of 1:5000-diluted horseradish peroxidase (HRP)-labeled goat anti-duck IgY or IgA was applied to the wells. The plates were incubated for 1 h at 37°C and washed five times with PBST.

### Statistical Analysis

All statistics were performed using GraphPad Prism 5 software package (Graph Software, San Diego, CA). Numerical data were expressed as means ± SD and evaluated with a student *t*-test. Differences were considered significant at *P* < 0.05. The median lethal dose (LD50) was estimated using a profit analysis.

## Results

### Heterologous Expression of *P. multocida fur* Gene in *S*. Choleraesuis

**Figure 1:**
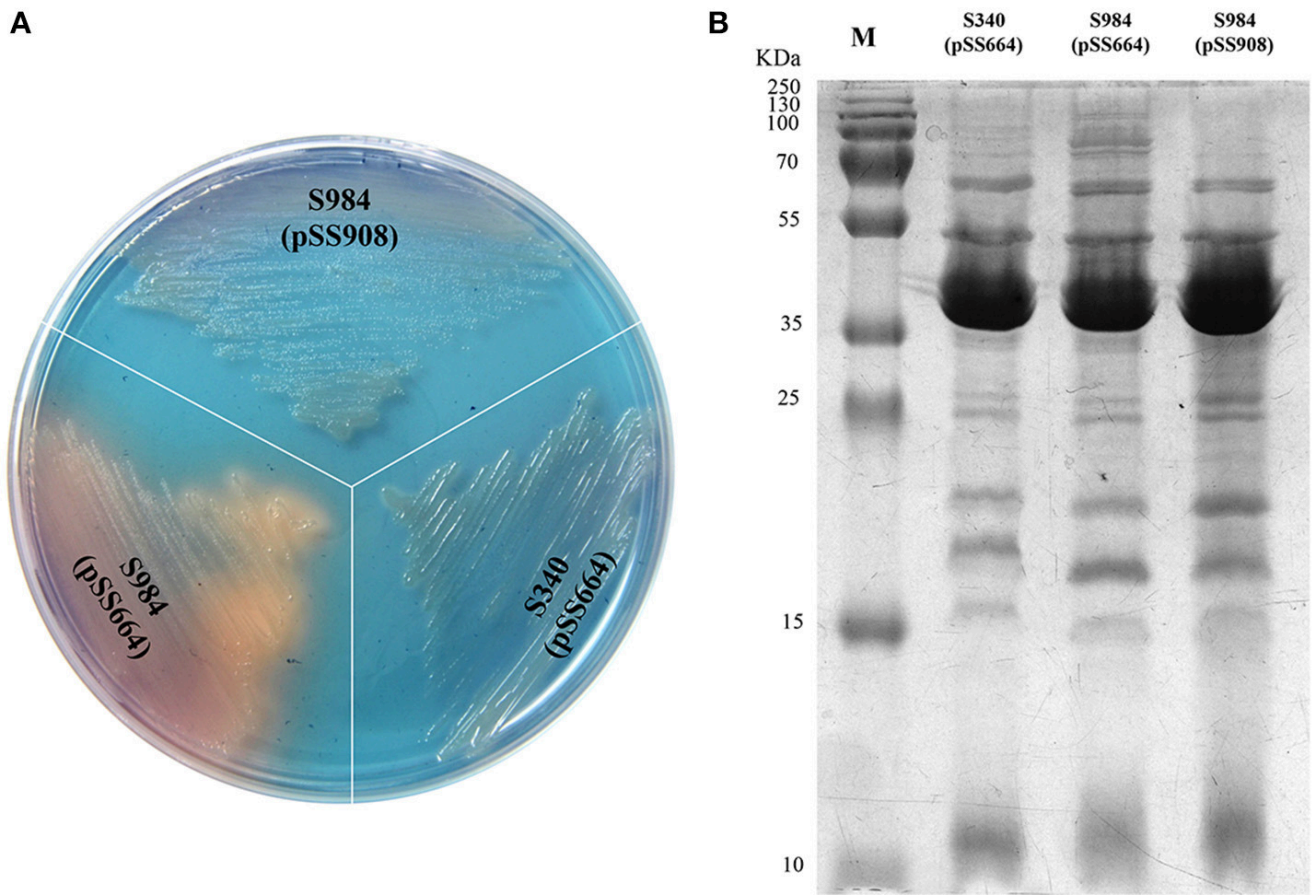
Identification of the *fur* gene of *P. multocida*. **(A)** Detection of the secreted siderophores. Three *S*. Choleraesuis strains (S340 harboring the control plasmid pSS664, S984 (Δ*fur*) harboring pSS664, and S984 harboring pSS908) were cultured on CAS plates, and the colony color was observed. **(B)** OMP profiles of *S*. Choleraesuis. OMPs were extracted from S340 (pSS664), S984 (Δ*fur*) (pSS664), and S984 (pSS908) and subsequently subjected to SDS-PAGE. Coomassie blue staining was applied to visualize the OMPs. M refers to the protein marker.

Based on our bioinformatics analysis, the *fur* gene of *P. multocida* Pm70 was 441 bp in length and shared no highly similar sequences with either *S*. Typhimurium *fur* (Gene ID 1252213) or *E. coli fur* (Gene ID 945295); however, the 441-bp sequence of the Pm70 *fur* gene was predicted to encode a 146-amino acid polypeptide, which showed 67% identity to both *S*. Typhimurium Fur (Protein ID, AAL19637.1) and *E. coli* Fur (Protein ID, CDZ19538.1). Thus, we proposed that this protein might perform the same functions as other Fur proteins. To further evaluate the functionality of *P. multocida* Fur, Δ*fur* mutants of *S*. Choleraesuis were complemented with the recombinant plasmid pSS908 carrying the *P. multocida fur* gene in the low-copy-number plasmid pSS664. *S*. Choleraesuis Δ*fur* (S984) was utilized for complementation assays (Table 1). The Δ*fur* mutant of *S*. Choleraesuis showed the synthesis and secretion of siderophores. The *P. multocida fur* gene complemented *S*. Choleraesuis Δ*fur*, repressing the synthesis and secretion of siderophores in an iron-dependent manner. Moreover, the OMP profiles of *S*. Choleraesuis (S340), *S*. Choleraesuis Δ*fur* (S984), and *S*. Choleraesuis Δ*fur* expressing *P. multocida* 0818 *fur* from the plasmid pSS908 were shown. As shown in Figure 1A, the *S*. Choleraesuis *fur* mutant (S984) harboring pSS664 displayed a yellow-orange colony, whereas the *S*. Choleraesuis parent strain (S340) harboring pSS664 and *S*. Choleraesuis Δ*fur* expressing *P. multocida* 0818 *fur* from the plasmid pSS908 displayed no changes in appearance. *S*. Choleraesuis Δ*fur* with the *P. multocida fur* gene and the *S*. Choleraesuis parent strain expressing vector pSS664 exhibited a minor difference of protein bands above 70 kDa compared with *S*. Choleraesuis Δ*fur* with vector pSS664 (Figure 1B).

### Construction and Characterization of *fur* Mutants

**Figure 2:**
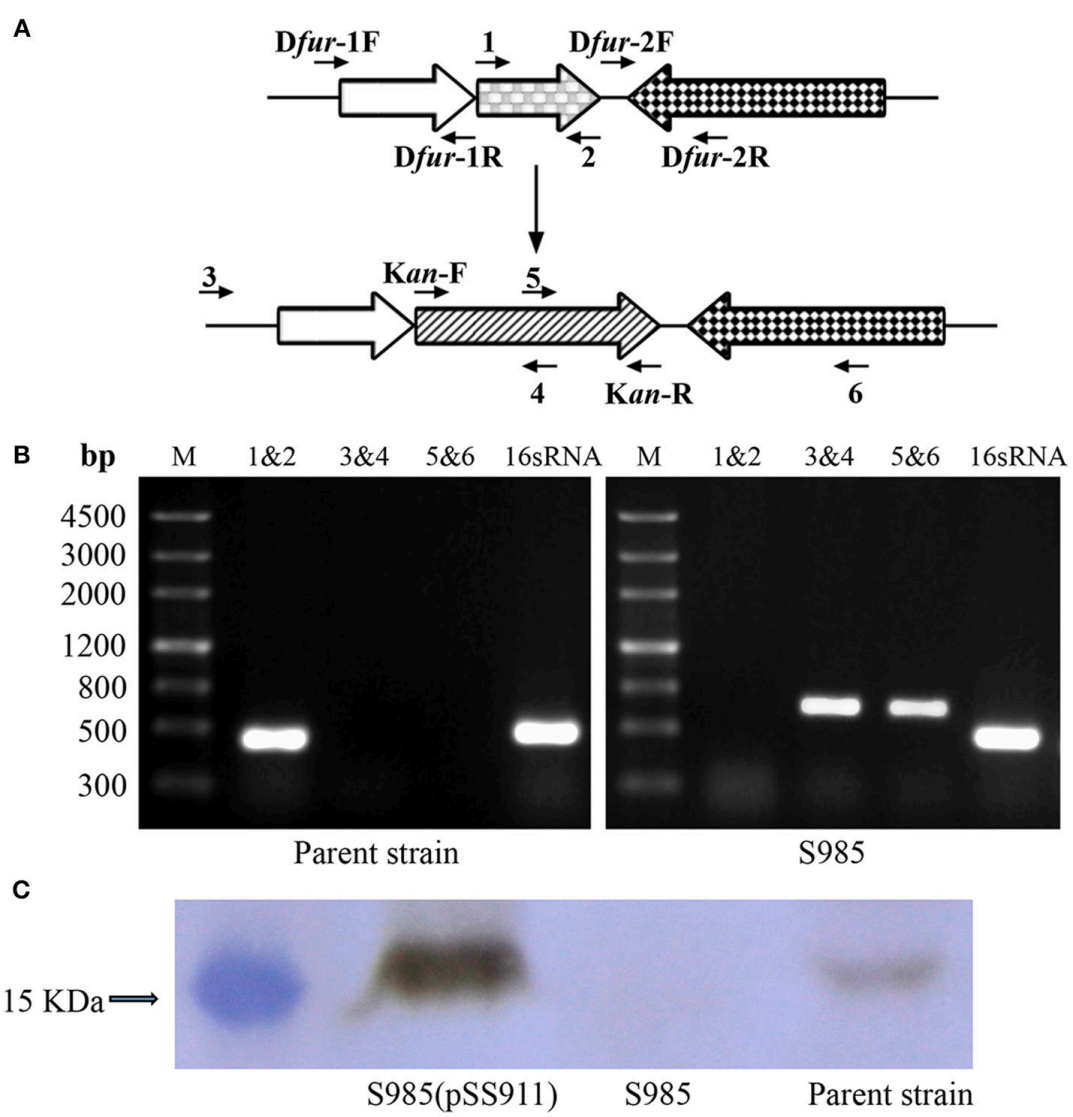
Construction of the Δ*fur* mutant in *P. multocida* strain 0818. **(A)** Schematic strategy for deletion of the target *fur* gene (PM0352). The targeted *fur* gene of *P. multocida* 0818 was replaced with a KanR cassette through homologous recombination. Primers 1, 2, 3, 4, 5, and 6 were designed for the selection and characterization of the mutant clones. **(B)** Identification of the constructed Δ*fur* mutant. The parent strain and S985 (Δ*fur*) were identified using three primer pairs: 1&2, 3&4, and 5&6. M refers to the DNA marker; 16S RNA denotes amplification of the positive control gene in both strains. **(C)** Detection of Fur expression in the *P. multocida* strains. The parent strain, S985 (Δ*fur*), and S985 (pSS911) were harvested at an OD_600_ of 1.0 following growth in BHI media, and the expression of Fur was subsequently detected in these strains using the mouse anti-Fur antibody via western blotting.

Construction of the *P. multocida* Δ*fur* mutant was performed using the suicide T-vector pYA4278 through suicide plasmid-mediated homologous recombination. The genotype was confirmed via PCR using three pairs of primers: 1&2, 3&4, and 5&6 (Figure 2A and Table 2 for primer sequences). Primers 3&6 were both designed based on the sequences of the *P. multocida* genome outside of the homology arms of *fur*. A fragment of *fur* (PM0352) was amplified using primers 1&2 (441 bp). Primers 4&5 were designed to target the middle of the *KanR* cassette. The two DNA segments amplified using primer pairs 3&4 and 5&6 (~650 bp) were present in the Δ*fur* mutant but not in the parent strain (*P. multocida* 0818). In contrast, the complete *fur* sequence (1&2) was present in the parent strain but not in the Δ*fur* mutant. The positive control 16S RNA sequence (501 bp) was amplified from both strains (Figure 2B). To further characterize the Δ*fur* mutant, the Fur protein of *P. multocida* was synthesized and purified, and a polyclonal mouse anti-Fur antibody was prepared for western blot assays. Fur was detected in the parent strain and the complementary S985 (pSS911) strain but not in S985 (Figure 2C), indicating that the *fur* gene was successfully deleted from S985.

### Phenotype Characterization of the *P. multocida fur* Mutant

**Figure 3:**
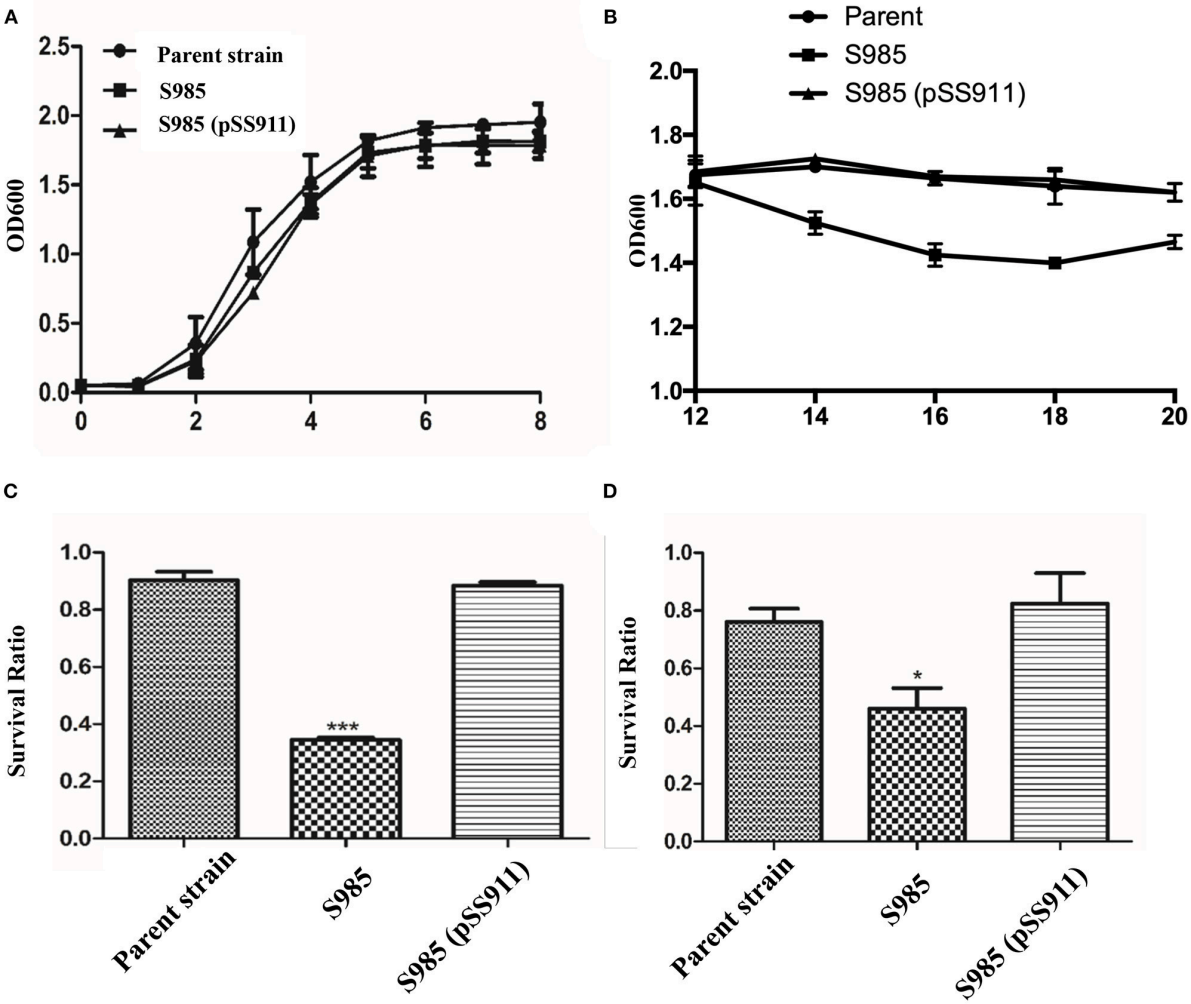
Phenotypic characterization of the *P. multocida* Δ*fur* mutant. **(A,B)** Growth curves of the parent strain and the Δ*fur* mutant. *P. multocida* was cultured in BHI broth supplemented with or without chloramphenicol, and the OD_600_ value of each strain was subsequently measured every 2 h over a period of 20 h, no significant differences for bacterial growth curves were observed. **(C,D)** Resistance to polymyxin B **(C)** or DOC **(D)**. The parent strain, S985 (Δ*fur*) and the complement strain S985 (pSS911) were cultured in BHI broth with or without 0.5 μg/ml polymyxin B and 250 μg/ml DOC for 1 h, and the survival ratio of each strain was calculated as the mean CFUs of the treated group divided by the mean CFUs of the untreated group. The data in **(C,D)** are expressed as the means ± SD analyzed at significance levels of 0.05 (*) and 0.001 (***).

To evaluate the influence of *fur* deletion in *P. multocida*, the growth rates, OMP profiles, and serum complement sensitivity of the parent strain (*P. multocida* 0818), S985 (*P. multocida* Δ*fur*), and S985 (pSS911) were determined. The parent strain, S985 (*P. multocida* Δ*fur*), and S985 (pSS911) exhibited similar and typical growth and proliferation patterns in a normal nutrient-rich (fresh BHI medium) environment (0-8 h), consisting of a (plateau) lag phase (0–2 h), a logarithmic phase (2–6 h), and a stationary phase (6–8 h; Figure 3A). However, when the iron ions in the medium were depleted, the S985 (*P. multocida* Δ*fur*) curve displayed a decline of growth due to the lack of effective iron control (12–14 h; Figure 3B). Both the parent strain and S985 (pSS911) exhibited similar, typical growth rates in both untreated and heat-treated healthy duck serum; however, S985 (*P. multocida* Δ*fur*) was rapidly killed in untreated serum but grew in heat-treated serum. We also observed that S985 did not grow to the extent of the parent and complemented strains in heat-treated serum (Table 3).

**Table 3:** Serum bactericidal assay.

**Strains**	**Serum heat treatment**	**Growth rate^**a**^**
*P. multocida* 0818	–	160 ± 47
	+	153 ± 28
S985	–	1 ± 0.4^b^
	+	31 ± 12
S985 (pSS911)	–	142 ± 43
	+	165 ± 38

a *The data represent the means and SD of three independent experiments*.

b *The difference in sensitivity between S985 in heated or unheated serum was determined to be significant (p < 0.01)*.

Alterations in the cell membrane protein profiles might reduce bacterial resistance to bile salts and cationic antimicrobial peptides; therefore, we examined the susceptibility of S985 (*P. multocida* Δ*fur*) to polymyxin B and DOC. The Δ*fur* mutant was highly sensitive to polymyxin B and DOC. After treatment with polymyxin B or DOC, the survival ratio of strain S985 (*P. multocida* Δ*fur*) was significantly lower than that of both the parent strain and the complement strain, S985 (pSS911) (Figures 3C,D).

**Figure 4:**
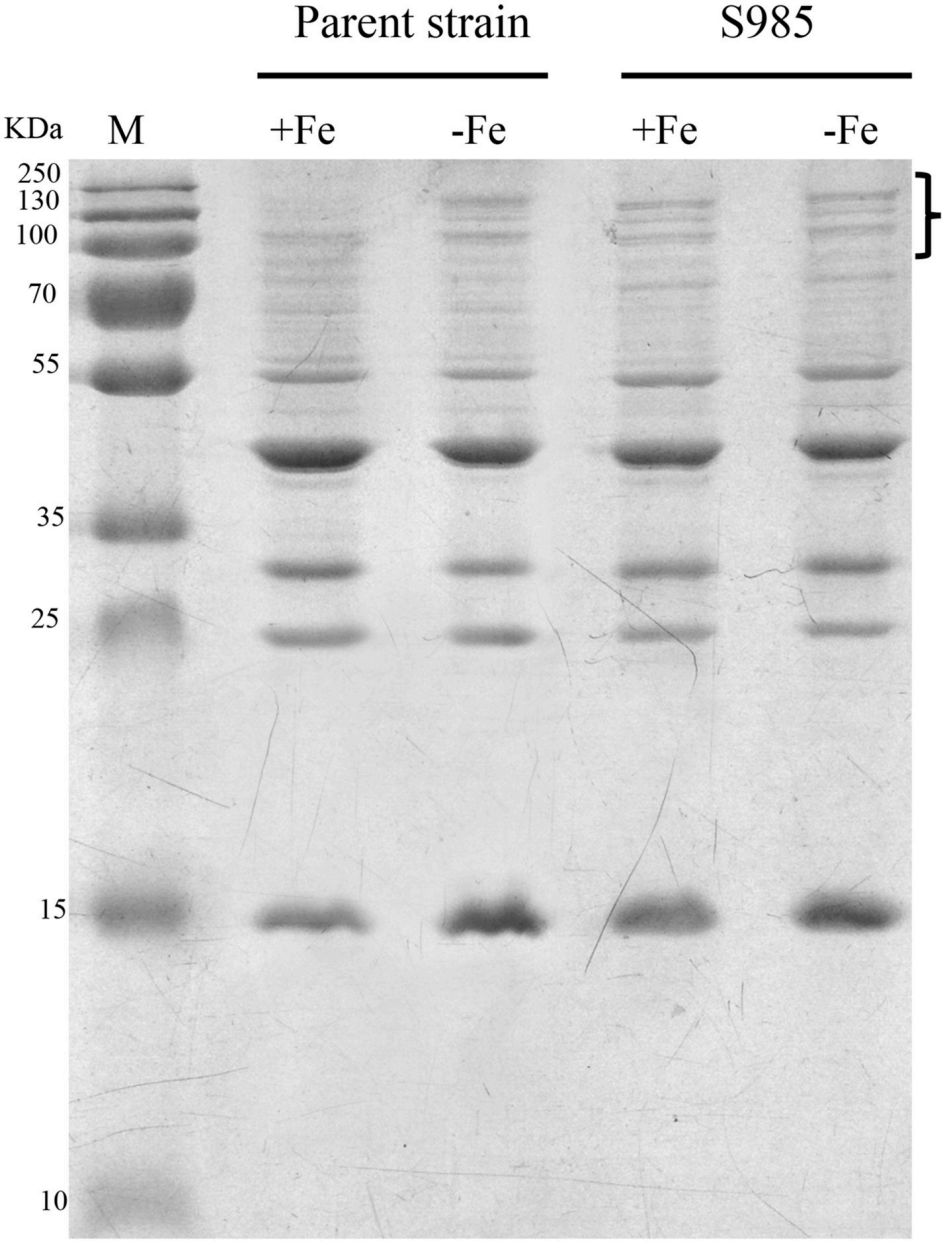
Fur-regulated IROMPs in *P. multocida*. Outer membrane profiles of *P. multocida. P. multocida* strain grown under iron-rich (+Fe, BHI broth) and iron-limited (-Fe, BHI broth plus 100 μM 2,2′-dipyridyl, DIP) conditions.

Wild-type *P. multocida*, grown under iron-limited conditions, and the *P. multocida* S985 *fur* mutant strain, grown under normal or iron-deficient conditions, both showed increased abundance of OMPs over 70 kDa (Figure 4), suggesting that these proteins are iron-Fur dependent and regulated by Fur or are associated with iron uptake.

### Colonization of the *P. multocida* Parent Strain and the Δ*fur* Mutant Strain

**Figure 5:**
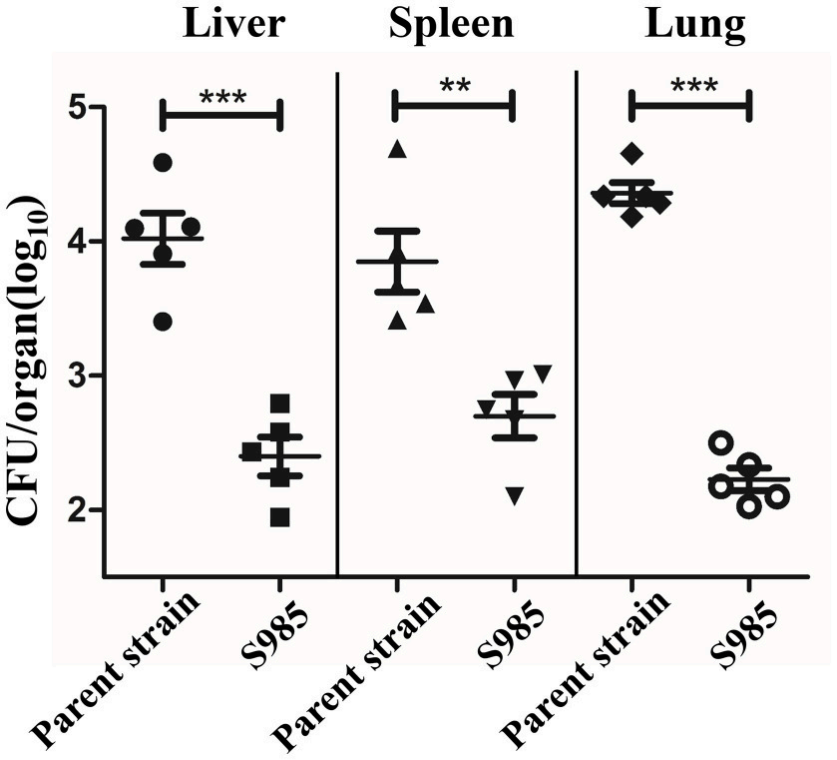
Colonization of the *P. multocida* parent strain and the Δ*fur* mutant strain. Colonization of Sheldrake duck tissues (*n* = 5) by the *P. multocida* parent strain and S985 (Δ*fur*) after 2 days of oral inoculation with 10^8^ CFUs of the respective strain. Each point represents one duck. The data are expressed as the means ± SD analyzed with significance levels set to 0.01 (**) and 0.001 (***).

We evaluated the colonization of the liver, spleen, and lungs of ducks 2 days after the oral inoculation with *P. multocida* Δ*fur* compared with the wild-type. We observed that the colonization levels of *P. multocida* Δ*fur* in the liver, spleen, and lungs were all lower than those of the wild-type (Figure 5). Interestingly, for the parent strain, the level of colonization in the lungs was higher than those in the liver and spleen; however, for the *fur* mutant strain, the bacterial load in the lungs was lower compared with those in the liver and spleen.

### Virulence of the *P. multocida* Δ*fur* Mutant in the Duck Host

To determine the effects of the *P. multocida* Δ*fur* mutant on virulence, the LD_50_ of the parent strain and *fur* mutant strain were evaluated in Sheldrake ducks, the natural host of *P. multocida* 0818. *P. multocida* Δ*fur* administered orally was attenuated. The LD_50_ of the Δ*fur* mutant was 1.65 × 10^8^ (Table 4), which represented an approximate 146-fold increase in the LD_50_ compared to that of the wild-type strain, which had an LD_50_ of 1.13 × 10^6^.

**Table 4:** Determination of the LD_50_ of *P. multocida* 0818 and the Δ*fur* mutant.

**Route**	**Strains**	**Challenge dose (CFU) and survival**				**LD_**50**_ (CFUs)**
		**10^**5**^**	**10^**6**^**	**10^**7**^**	**10^**8**^**	
Oral	*P. multocida* 0818	6/10	4/10	2/10	0/10	1.13 × 10^6^
	S985 (Δ*fur*)	15/15	13/15	11/15	8/15	1.65 × 10^8^

### Immune Protection of *P. multocida* Δ*fur* Mutants in the Duck Host

**Figure 6:**
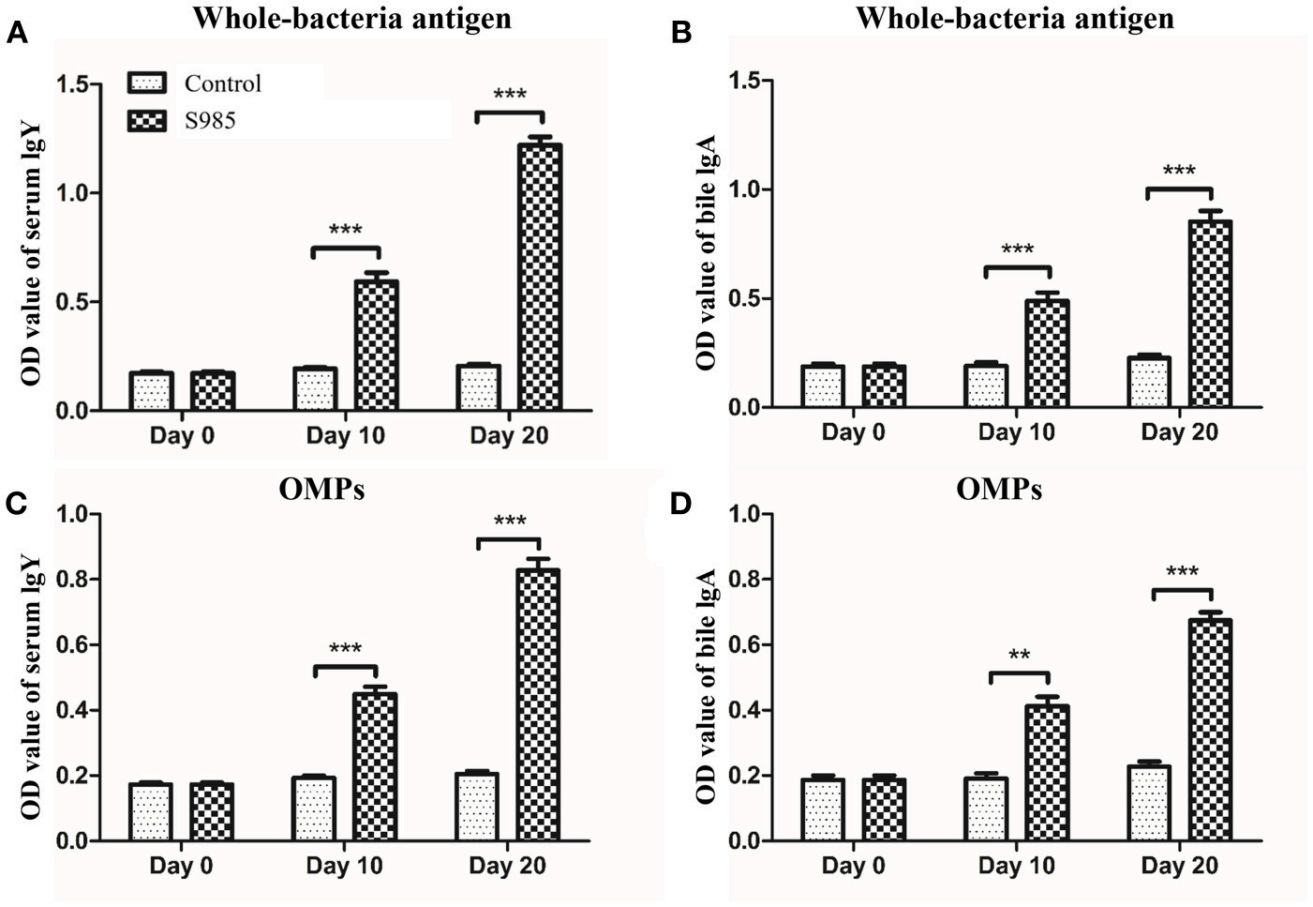
Antibody responses induced by the Δ*fur* mutant in ducks. Ducks (*n* = 5) were orally immunized with the Δ*fur* mutant strain twice at an interval of 10 days. Subsequently, the responses of serum IgY specific to whole-bacterial antigens **(A)** and OMPs **(C)** and the responses of bile IgA specific to whole-bacterial antigen **(B)** and OMPs **(D)** were measured using indirect ELISA. The data are expressed as the means ± SD analyzed with significance levels set to 0.01 (**) and 0.001 (***).

Ducks orally immunized with *P. multocida* Δ*fur* survived an oral challenge with 500 × the LD_50_ of wild-type *P. multocida* 0818 for 10 days after the second immunization, whereas all control ducks died within 2 days (Table 5). The whole-bacterial antigen- and OMP-specific responses of the serum IgY and bile IgA levels of ducks in samples prepared on day 0 prior to immunization and days 10 and 20 post-immunization were measured by indirect ELISA. As shown in Figure 6, no specific serum IgY or bile IgA responses to *P. multocida* antigens or OMPs were present 1 day prior to immunization. In contrast, the immunized group exhibited strong stimulation of serum IgY and bile IgA antibodies against whole-bacterial antigens and demonstrated significant IgY and IgA titres in the blood and bile, respectively. Similar to the results for whole-bacterial antigens, OMP antigen-specific serum IgY and bile IgA responses were stronger in the vaccinated group than in the control group.

**Table 5:** Protection rate conferred by the Δ*fur* mutant.

**Group**	**Immunization**	**Challenge**	**Survival**	**Protection**
Immune group	1 × 10^5^ CFU of S985 (Δ*fur*)	5 × 10^8^ CFU of *P. multocida* 0818	31/50	62%
Control	PBS	5 × 10^8^ CFU of *P. multocida* 0818	0/15	0

## Discussion

*P. multocida* causes acute infectious zoonotic diseases in humans and animals, including fowl cholera in birds, atrophic rhinitis in pigs, haemorrhagic septicaemia in cattle, snuffles in rabbits, and wound abscesses or meningitis in humans (33). These diseases pose significant threats to the food industry. Global regulators modulate the transcription and expression of hundreds of genes, including virulence factors. Therefore, studies of *P. multocida* global regulators provide a better understanding of immune escape mechanisms in hosts and facilitate the identification of toxins or released toxic by-products as well as other molecular mechanisms underlying host-pathogen interactions, providing significant aid to vaccine development. In-depth analyses performed in recent years have revealed several global regulators in *P. multocida*, such as PhoP/PhoQ (21), Crp (cAMP receptor protein) (28), Fis (nucleoid-associated protein) (34), and FnrP (35, 36). Fur is another global regulator that regulates the expression of iron acquisition genes and many other virulence genes.

Fur acts as either a repressor or an activator to regulate gene expression by binding as a Fe^2+^-Fur complex to promoter regions (Fur boxes) upstream of its target genes. Fe^2+^-Fur activation might occur directly, creating a competition for DNA binding sites with other inhibitors (37). For example, the bacterial ferritin gene *bfrB* in *Pseudomonas aeruginosa* and the *hilD* virulence factor in *S*. Typhimurium are both activated by Fe^2+^-Fur (38, 39). The indirect activation of Fur might also occur. Fe^2+^-Fur negatively regulates certain small regulatory RNAs. RyhB, a small non-coding RNA (sRNA), triggers the mRNA degradation through base pairing via RNase E and RNase III in *E. coli* (40). In *P. multocida*, a DNA microarray analysis showed that the transcriptional regulation of 174 genes was altered under iron-limited growth conditions, which includes virulence genes involved in iron uptake from the environment (41). In the present study, profiling of *P. multocida* outer membrane proteins revealed that the OMP bands of parent strains grown in iron-deficient environments were similar to the OMP bands of Δ*fur* mutant strains grown under iron-limited or iron-sufficient conditions (Figure 3), suggesting that the expression of iron-regulated outer membrane proteins (IROMPs) is regulated by Fur and iron-restricted conditions.

As a global regulator, Fur plays essential roles in virulence. The absence or impairment of the *fur* gene leads to the attenuation of bacterial virulence. For example, Δ*fur* mutation in *Campylobacter jejuni* has a significant effect on its colonization of the gastrointestinal tract in chickens (42). Fur proteins of *N. meningitidis* and *N. gonorrhoeae* are involved in disease pathogenesis(43). In the present study, we observed that the virulence of S985 (Δ*fur*) decreased 146-fold after oral inoculation, indicating that the Fur protein plays a role in the regulation of virulence factors of *P. multocida* in the host iron-limiting environment. However, the decreased virulence observed with the *P. multocida* Δ*fur* mutant strain was markedly less pronounced than in other common strains, such as *Edwardsiella ictaluri* (6) and *S*. Typhimurium (44). Thus, other genes of *P. multocida* might replace the *fur* iron regulation function to minimize adverse effects in *fur*-deleted strains. Moreover, the virulence of the *P. multocida* 0818 Δ*fur* mutant strain differed from that of another *P. multocida* Δ*fur* mutant strain constructed in 2001. The 2001 *P. multocida* Δ*fur* mutant strain exhibited the same virulence as its *P. multocida* 0818 parent strain (LD_50_ = 5 CFU per animal) when the bacteria were intraperitoneally inoculated in Swiss mice (45). Similar results has been also observed for *S*. Typhimurium Δ*fur* mutant when inoculated intraperitoneally and orally into the mouse model (44), indicating that the infective route, the environmental conditions, and the selected host affect the virulence of the bacteria.

Live oral vaccines encounter gastric acid in the stomach, the weak alkaline environment, bile salt (e.g., DOC), and antimicrobial peptides (e.g., polymyxin B) in the intestinal fluid, en route to the site of host cells targeted for colonization. We observed the increased susceptibility of S985 (Δ*fur*) to polymyxin B and DOC (data not shown), consistent with other observations that the *fur* mutant strains of *S*. Typhimurium and the uropathogenic *E. coli* strain CFT073 show significantly greater sensitivity to oxidative stress compared to their wild-type parents (46, 47). S985 (Δ*fur*) died more rapidly under insufficient nutrient conditions when grown in BHI over 12 h (Figure 3B) and showed a more obvious decrease in the colonization of these organs (Figure 4). Although *fur* mutant strains exhibited certain defects in host organs, the bacterial load and persistence of S985 (Δ*fur*) in host organs was sufficient to induce humoral immune responses (Figure 4) and provided sufficient protection against a lethal dose challenge of wild-type *P. multocida*.

In addition to achieving adequate homologous protection, cross-protection has become a criterion for evaluating vaccine quality. Many researchers have suggested that the growth of *P. multocida in vivo*, particularly in iron-restricted environments, results in iron-regulated OMPs that are adequate cross-protective antigens (48–50). The *P. multocida fur* mutant continuously synthesized IROMPs, resulting in exposure to more epitopes that continuously stimulate the host's immune system to produce antibodies (Figures 6A–D). However, these high levels of IgG are not fully protective under challenges with high doses of virulent *P. multocida*. Although the *P. multocida* Δ*fur* mutant strain is not a highly efficient and safe live-attenuated vaccine, *fur* is a global regulatory gene that controls the expression of virulence factors. Accordingly, the *fur* gene might be deleted together with other genes, such as the regulators *phoP* and *crp* or *fis* (essential for capsule production) (34) to increase attenuation and enhance cross-protection effects and immunogenicity of the vaccine.

## Author Contributions

QK and QL designed the experiments and analyzed the data. YH and PL carried out the experiments. QL, YH and QK wrote the paper.

### Conflict of Interest Statement

The authors declare that the research was conducted in the absence of any commercial or financial relationships that could be construed as a potential conflict of interest.
